# Oral Fosfomycin for the Treatment of Acute and Chronic Bacterial Prostatitis Caused by Multidrug-Resistant *Escherichia coli*

**DOI:** 10.1155/2018/1404813

**Published:** 2018-01-30

**Authors:** George G. Zhanel, Michael A. Zhanel, James A. Karlowsky

**Affiliations:** Department of Medical Microbiology and Infectious Diseases, Max Rady College of Medicine, University of Manitoba, Winnipeg, MB, Canada

## Abstract

Acute and chronic bacterial prostatitis in outpatients is commonly treated with oral fluoroquinolones; however, the worldwide dissemination of multidrug-resistant (MDR) *Escherichia coli* has resulted in therapeutic failures with fluoroquinolones. We reviewed the literature regarding the use of oral fosfomycin in the treatment of acute and chronic prostatitis caused by MDR *E. coli*. All English-language references on PubMed from 1986 to June 2017, inclusive, were reviewed from the search “fosfomycin prostatitis.” Fosfomycin demonstrates potent in vitro activity against a variety of antimicrobial-resistant *E. coli* genotypes/phenotypes including ciprofloxacin-resistant, trimethoprim-sulfamethoxazole-resistant, extended-spectrum *β*-lactamase- (ESBL-) producing, and MDR isolates. Fosfomycin attains therapeutic concentrations (≥4 *μ*g/g) in uninflamed prostatic tissue and maintains a high prostate/plasma ratio up to 17 hours after oral administration. Oral fosfomycin's clinical cure rates in the treatment of bacterial prostatitis caused by antimicrobial-resistant *E. coli* ranged from 50 to 77% with microbiological eradication rates of >50%. An oral regimen of fosfomycin tromethamine of 3 g·q 24 h for one week followed by 3 g·q 48 h for a total treatment duration of 6–12 weeks appeared to be effective. Oral fosfomycin may represent an efficacious and safe treatment for acute and chronic prostatitis caused by MDR *E. coli*.

## 1. Introduction

Acute and chronic bacterial prostatitis is difficult to treat as few antimicrobials attain therapeutic concentrations in the prostate [1, 2]. In terms of orally available antimicrobials, the *β*-lactams demonstrate limited penetration into the prostate [1, 2]. The tetracyclines achieve sufficient concentrations in the prostate, but extensive resistance limits their use [1, 2]. Trimethoprim-sulfamethoxazole (TMP-SMX) has been used successfully to treat bacterial prostatitis, due to sufficient prostate penetration, but resistance also has limited its use. The pharmacokinetic (PK) and pharmacodynamic (PD) properties of orally administered fluoroquinolones, including their broad-spectrum bactericidal activity covering common pathogens associated with prostatitis and good prostate penetration, have made them the agents of choice for the management of acute and chronic bacterial prostatitis for the past 25 years [1, 2].

*Escherichia coli* continues to be the most common cause of uncomplicated and complicated urinary tract infections as well as acute and chronic bacterial prostatitis, although other organisms including enterococci species are increasing [1–6]. Since 2000, progressive increases in fluoroquinolone resistance among clinical isolates of *E. coli* have been reported; more recently, the emergence and proliferation of a dominant multidrug-resistant (MDR) subclone of sequence type 131 (ST131) has contributed to increasing fluoroquinolone resistance [3, 4]. ST131 is also associated with the spread of extended spectrum *β*-lactamase- (ESBL-) producing *E. coli*, primarily carrying CTX-M-14 and CTX-M-15 which confer resistance to cephalosporins, as well as resistance determinants for TMP-SMX and tetracyclines [3–6]. We have recently reported that 76.3% of fluoroquinolone-resistant and 56.1% of ESBL-producing isolates of *E. coli* collected across Canada were ST131 [3, 4]. As these ESBL-producing MDR *E. coli* continue to spread not only within Canada but around the globe, clinicians and researchers worry that more fluoroquinolone treatment failures will be reported in patients with bacterial prostatitis [7]. Disturbingly, some of the MDR ESBL-producing *E. coli* (and *Klebsiella* spp.) are growing becoming resistant to the carbapenems, even further complicating the treatment of acute and chronic prostatitis [3].

Fosfomycin has been available to physicians in many European countries as well as Japan, South Africa, and Brazil, in both oral and parenteral formulations, for up to four decades [8–11]. Oral fosfomycin first entered the Canadian and US markets in 1997 but was withdrawn in Canada several years later due to lack of use [8]. It was recently reintroduced in Canada and is indicated for the treatment of acute uncomplicated cystitis in adult women infected with susceptible isolates of *E. coli* and *Enterococcus faecalis* [8]. Our research group and others have recently reported that fosfomycin demonstrates potent in vitro activity versus antimicrobial-resistant *E. coli* including ESBL-producing, AmpC-producing, and MDR isolates [6, 9–11]. In addition, its ability to attain therapeutic concentrations in prostatic fluids or secretions along with a favourable safety profile has resulted in clinicians asking about its potential role for the treatment of acute and chronic bacterial prostatitis in the setting of MDR *E. coli* isolates when fluoroquinolones cannot be used [6, 9–11]. This review seeks to provide an overview of the potential role of oral fosfomycin in the treatment of acute and chronic bacterial prostatitis caused by MDR *E. coli*, which includes a comprehensive review of available clinical data.

## 2. Current Antimicrobial Treatment for Acute Bacterial Prostatitis

Most patients (∼85%) diagnosed with acute bacterial prostatitis (ABP-National Institutes of Health type I) can be successfully treated as outpatients with oral antimicrobials [12]. Hospitalization and intravenous antimicrobial therapy may be warranted in patients with ABP who have failed outpatient management are systemically ill, are unable to tolerate oral intake, or present with urinary retention [12]. Empiric outpatient antimicrobial therapy should commence immediately after clinical diagnosis with subsequent optimization of treatment based on urine and blood culture pathogen/susceptibility results [2]. Clinicians should consider local antimicrobial resistance trends prior to empiric treatment, especially with the increasing proliferation of ESBL-producing MDR *E. coli*, and the increasing role of other organisms including enterococci species [1, 3]. Oral antimicrobial treatment courses of 2–4 weeks duration are generally sufficient to provide microbiological and clinical cure [13]. A urine culture one week following completion of antimicrobial therapy indicating bacterial eradication is suggested to confirm microbiological cure [13].

Empiric oral antimicrobial treatment regimens for ABP vary depending on age and sexual activity of patients [12]. Fluoroquinolones, including ciprofloxacin and levofloxacin, are the preferred oral agents for the treatment of ABP [1]. Due to resistance, some clinicians prefer to use combination treatment [1, 2]. Alternative oral agents may also be effective if they can penetrate acutely inflamed prostatic tissue and attain therapeutic concentrations at the site of infection [2]. If hospitalization is required, a wide range of intravenous agents can be used including fluoroquinolones, third- and fourth-generation cephalosporins, piperacillin-tazobactam, carbapenems, and aminoglycosides [12]. In patients where the risk of a sexually transmitted infectious pathogen is low, oral ciprofloxacin 500 mg BID (twice daily) for 10–14 days or oral levofloxacin 500–750 mg OD (once daily) for 10–14 days is recommended [12]. TMP-SMX 160/800 mg BID × 10–14 days is an oral alternative to fluoroquinolones [2]. Regimens covering *Neisseria gonorrhoeae* and *Chlamydia trachomatis* are recommended in sexually active men under 35 years of age and men over 35 years of age exhibiting high-risk sexual behaviour [12]. For patients satisfying these criteria, intramuscular ceftriaxone or oral cefixime, followed by doxycycline is recommended [12].

## 3. Current Antimicrobial Treatment for Chronic Bacterial Prostatitis

Approximately 10% of patients diagnosed with ABP will develop chronic bacterial prostatitis (CBP-National Institutes of Health type II) [13]. CBP is characterized by recurrent urinary tract infections (UTIs) due to persistence of the same causative pathogen resulting in unresolved urogenital symptoms [13]. Similar symptoms are reported in cases of acute and chronic bacterial prostatitis including dysuria, urgency, and perineal pain; however, patients with CBP are generally afebrile unlike patients with ABP [2, 13]. Individuals with CBP typically cycle between symptomatic and asymptomatic periods for a prolonged period of time (>3 months) despite ongoing infection [1].

Treatment for CBP is often much more problematic than ABP as reflected in high rates of recurrence (25–50%) [13]. Achieving therapeutic concentrations of antimicrobial agents at the site of infection is a major limitation to the effective treatment of CBP with oral antimicrobial agents [1]. Unlike ABP, prostatic tissue in patients with CBP may be inconsistently inflamed despite persistent infection. Only agents with suitable pharmacological properties can cross prostatic capillary endothelium and attain therapeutic concentrations in prostatic epithelium [13]. Agents possessing small molecular size, high lipid solubility, a low degree of ionization, high p*K*_a_ values, and low protein binding are generally favourable for penetration into prostatic fluids or secretions [1]. Fluoroquinolones, sulfonamides, macrolides, and tetracyclines generally exhibit these pharmacological properties and have demonstrated clinical efficacy in the treatment of CBP [13]. Oral antimicrobial courses of ≥4 weeks are considered optimal for CBP; oral therapy of up to 6 weeks in duration is sometimes used [13]. Treatment should commence after obtaining urine/blood culture results and ideally be tailored to pathogen/susceptibility data [1]. Repeat courses of antimicrobials are discouraged for fear of generating antimicrobial-resistant strains, although some evidence suggests that long-term low-dose courses of antimicrobials may minimize symptomatic recurrences and be of particular benefit to patients with abnormal genitourinary pathology including prostatic calculi [1, 13].

Fluoroquinolones are the most commonly used first-line oral antimicrobials for the treatment of CBP due to their superior penetration into uninflamed prostatic fluids or secretions (10–50% of serum concentrations) [2, 14, 15]. A comparative study published by Perletti et al. indicated that ciprofloxacin, levofloxacin, lomefloxacin, ofloxacin, and prulifloxacin all demonstrated comparable clinical and microbiological efficacy in the treatment of CBP [16]. Ciprofloxacin at a dose of 500 mg BID for 4–6 weeks or levofloxacin at a dose of 500 mg OD for 4–6 weeks are commonly cited oral regimens for the treatment of CBP [2, 14]. TMP-SMX is also a commonly used oral agent for the treatment of CBP; however, it is widely recognized to be less effective than fluoroquinolone therapy due to diminished penetration into prostatic fluids or secretions and higher rates of resistance [1, 14]. Doxycycline is an alternative second-line agent; however, extensive tetracycline resistance has greatly diminished its efficacy in the treatment of CBP in the last decade [1, 2, 14]. Macrolides such as azithromycin or clarithromycin are recommended in cases of CBP caused by atypical bacterial pathogens such as *Chlamydia trachomatis* [16].

## 4. Antimicrobial Resistance in *E. coli*

*E. coli* is the most common causative pathogen of acute and CBP [1, 2]. Epidemiological data indicates that *E. coli* is responsible for 50–80% of bacterial prostatitis cases [1]. Most remaining cases are caused by other Gram-negative bacilli, including *Enterobacteriaceae* (e.g., *Klebsiella* spp. and *Proteus* spp.) and *Pseudomonas aeruginosa* [1]. *Enterococcus* spp. comprise a minority (5%–10%) of bacterial prostatitis cases, while atypical pathogens (e.g., *C. trachomatis*, *N. gonorrhoeae*, *Ureaplasma urealyticum*, *Mycoplasma genitalium*, and *Trichomonas vaginalis*), including those spread by sexual contact, are also infrequently the cause of bacterial prostatitis [2].

We have recently reported a significant increase in the proportion ESBL-producing *E. coli* in Canadian hospitals [3, 4]. Our research group and others have identified that the proliferation of ESBL-producing *E. coli* in Canada has been largely associated with the spread of a pandemic clone, *E. coli* O25b:H4 ST131 [3, 17]. Our data further demonstrated that >75% of ESBL-producing *E. coli* exhibited a MDR phenotype, hence, establishing a strong association between the ESBL genotype and the MDR phenotype [3]. We reported an increase in the proportion of ESBL-producing *E. coli* from 3.4% to 7.1% and an increase in the frequency of MDR among ESBL-producing *E. coli* of 77.4% to 82.6% over a 5-year period [3]. Concomitant resistance to fluoroquinolones, TMP-SMX, tetracyclines, and amoxicillin-clavulanate is common in ST131 *E. coli*, particularly in association with the ESBL genes CTX-M-14 and CTX-M-15 [3, 18, 19]. The increasing prevalence of antimicrobial-resistant phenotypes among *E. coli* in Canada has led to growing concern about the efficacy of empirical treatments for urinary tract infections including acute cystitis, pyelonephritis, and more recently, acute and CBP [8, 20].

The difficulty in treating bacterial prostatitis, especially chronic cases, can be in part attributed to the high rates of resistance to empirical agents [21]. High rates of fluoroquinolone resistance, TMP-SMX resistance, and MDR *E. coli* have played an increasing role in the poor prognosis of patients with bacterial prostatitis [3–6, 21]. Recent in vitro data from our national CANWARD surveillance study from 2007 to 2015, which included 1,207 *E. coli* isolates across 15 hospitals in Canada, indicated ciprofloxacin and TMP-SMX resistance rates of 18.9% and 25.0%, respectively, as shown in Table 1 [4]. Our group concluded that current fluoroquinolone and TMP-SMX resistance rates in *E. coli* exceed limits that, in some cases, no longer support their empirical use in therapy [6].

Fosfomycin has growingly received attention for the treatment of bacterial prostatitis after demonstrating clinical efficacy in the treatment of acute uncomplicated cystitis and promising in vitro activity against ESBL-producing MDR *E. coli* [6, 8]. Fosfomycin demonstrates potent in vitro activity against a variety of resistant phenotypes including ESBL-producing, ciprofloxacin-resistant, TMP-SMX-resistant, and MDR isolates of *E. coli* (Table 1). Overall, *E. coli* susceptibility to fosfomycin was reported to be 99.2% with susceptibility rates of 99.7%, 96.1%, 95.1%, and 100% for TMP-SMX-resistant, ciprofloxacin-resistant, ESBL-producing, and MDR isolates (Table 1) [4]. Zhanel et al. [8] and Mezzatesta et al.[20] reported similar data and corroborated our conclusion that fosfomycin's in vitro activity indicates that it may be a viable empirical therapy for uncomplicated UTIs, in the setting of extensive fluoroquinolone and TMP-SMX resistance in *E. coli* . These promising in vitro data, however, need to be assessed along with fosfomycin's pharmacological properties to determine its true potential for the treatment of acute and chronic prostatitis caused by MDR *E. coli*.

## 5. Fosfomycin Penetration into Prostatic Fluids or Secretions

Oral fosfomycin is typically administered using the fosfomycin tromethamine (FT) formulation due to superior bioavailability (∼40%) versus fosfomycin calcium (∼12%) [10]. Upon oral administration, FT is rapidly absorbed in the gut where it enters the bloodstream and dissociates, releasing fosfomycin as a free acid [8, 10]. Once in the blood, fosfomycin is distributed throughout the body to a variety of tissues and biological fluids including the kidneys, bladder, prostate, lungs, cerebrospinal fluid, bone, abscess fluid, and heart valves [8, 10]. Fosfomycin has a large volume of distribution (*V*_*d*_ of ∼2 L/kg) indicating extensive tissue/cellular penetration [22]. Fosfomycin's ability to successfully penetrate and achieve therapeutic concentrations in prostatic fluids or secretions is likely due to favourable pharmacological properties including small molecular size and low protein binding [1, 8, 10]. In addition, the high lipid solubility of fosfomycin is favourable for penetration into the lipid-rich parenchyma of the prostate [22].

**Figure 1:**
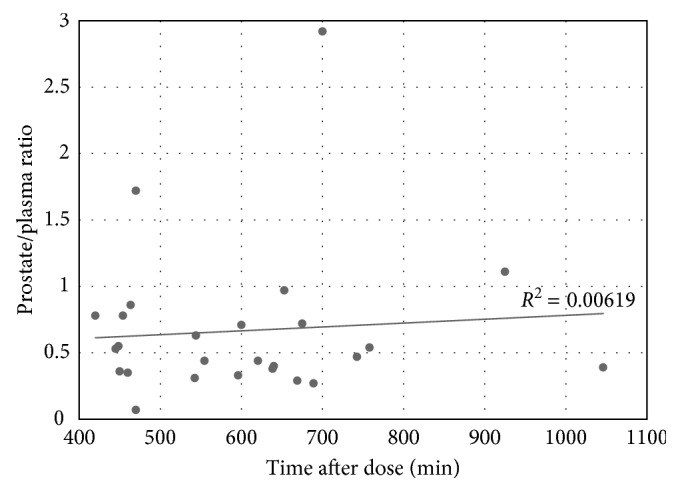
Prostate/plasma ratios after administration of a single 3 g dose of oral fosfomycin.

Few peer-reviewed studies have assessed fosfomycin's penetration into human prostatic fluids or secretions. Gardiner et al., published the first prospective human study measuring intraprostatic fosfomycin concentrations after administration of a single 3 g oral preoperative dose to patients undergoing transurethral resection of the prostate (TURP) [9]. Plasma, urine, and prostatic tissue biopsies from the transition and peripheral zones were obtained at single time points after commencement of TURP surgery for each of the 26 subjects (mean age, weight, and eGFR (estimated glomerular filtration rate) of 68 ± 9 years, 86.2 ± 13 kg, and 67 ± 12 mL/minute/1.73 m^2^, resp.) [9]. All 26 subjects in the study were undergoing treatment for benign prostatic hyperplasia and were otherwise healthy. Fosfomycin concentrations were measured using liquid chromatography-mass spectrometry (Table 2). The mean fosfomycin concentration in the transition zone was determined to be 8.30 ± 6.63 *μ*g/g (range, 0.56–26.05 *μ*g/g) measured at a mean postdose time of 598 ± 152 min [9]. The mean concentration in the peripheral zone was determined to be 4.42 ± 4.10 *μ*g/g (range, 0.17–18.06 *μ*g/g) measured at a mean postdose time of 608 ± 155 min [9]. The overall mean prostate concentration was reported to be 6.50 ± 4.93 *μ*g/g (range, 0.67–22.06 *μ*g/g) with a mean postdose measurement time of 602.9 ± 153.36 min [9]. Seventy percent of subjects demonstrated mean fosfomycin prostatic concentrations of ≥4 *μ*g/g at the time of measurement, indicating they achieved a concentration above the MIC_90_ (≥4 *μ*g/mL) of *E. coli* [8–10]. Average plasma concentrations were reported to be 11.42 ± 7.60 *μ*g/mL (range, 2.29–40.38 *μ*g/mL) measured at a mean postdose time of 565 ± 149 min [9]. Mean fosfomycin urine concentrations were 570.57 ± 418.40 *μ*g/mL (range, 47.99–1522.05 *μ*g/mL) measured at a mean postdose time of 581 ± 150 min. Data from each participant was used to determine the mean prostate/plasma ratio (0.67 ± 0.57; range, 0.07–2.92) as graphically represented in Figure 1 [9]. This study successfully demonstrated that fosfomycin is capable of reaching therapeutic concentrations in the prostate and maintains a high prostate/plasma ratio up to 17 hours after oral administration of a single dose [9]. For patients with acute and chronic bacterial prostatitis, inflammation of the prostate would likely enhance prostatic penetration of fosfomycin, and prostatic fluid or secretion concentrations would be expected to be even greater than those reported by Gardiner et al. [9].

Rhodes et al. assessed the optimal timing of prophylactic oral fosfomycin administration prior to TURP [23]. Plasma, peripheral zone, and transition zone fosfomycin concentrations were obtained from 26 subjects undergoing TURP following a single oral dose of 3 g of fosfomycin. Rhodes et al. reported that fosfomycin is likely to reach prostatic concentrations ≥ 4 *μ*g/g, when administered 1–4 hours prior to surgery [23]. Rhodes et al. reported that fosfomycin transition zone prostate concentrations exceeded 4 *μ*g/mL in 90% of the population between hours 1 and 9 after fosfomycin administration while peripheral zone prostate concentrations exceeded 4 *μ*g/mL in 70% of the population between hours 1 and 4. The authors concluded that oral fosfomycin should be administered 1–4 hours prior to prostate biopsy in order to achieve therapeutic concentrations in the prostate to prevent postoperative infection with *E. coli* [22].

## 6. Fosfomycin for the Treatment of Acute and Chronic Prostatitis

To our knowledge, a total of four publications (3 papers and one poster) have reported clinical data of oral fosfomycin therapy in the treatment of acute and chronic prostatitis (Table 3) [7, 22, 24, 25]. Los-Arcos et al. published a retrospective study in 2016 detailing 15 difficult-to-treat cases of CBP treated with oral fosfomycin [7]. The subjects (median age of 54 years) were selected based on the following inclusion criteria: diagnosis of CBP, failure of prolonged first-line antimicrobial therapy, and no possibility of successful fluoroquinolone or TMP-SMX treatment due to resistance, failure, or side effects. CBP diagnosis was determined when all four of the following criteria were satisfied: (i) ≥1 symptomatic occurrence of prostatitis of ≥4 weeks duration or ≥2 episodes of any duration in the preceding 12 months, (ii) active symptoms of prostatitis, (iii) absence of genitourinary abnormality as determined by urologic ultrasound on more than one occasion, and (iv) evidence of infection as determined by a positive Meares–Stamey test, positive semen culture, or ≥2 urine cultures with presence of the same pathogen ≥ 1 month apart. First-line antimicrobial therapy failure was defined by persistence of the same causative pathogen in cultures after treatment with ciprofloxacin at a dose of 500 mg/12 h for ≥4 weeks or TMP-SMX at a dose of 160 mg/800 mg/12 h for ≥6 weeks. All subjects in the study fulfilled the inclusion criteria and also fell within the clinical definition of CBP. *E. coli* was isolated as the causative pathogen in 14/15 (93.3%) subjects in the study. MDR (resistant to at least one agent in ≥3 antimicrobial drug-classes) *E. coli* accounted for 5/14 (35.7%) isolates. An ESBL phenotype was identified in 4/14 (28.6%) *E. coli* isolates, and 1/14 (7.1%) *E. coli* isolates was identified as an AmpC *β*-lactamase producer. A total of 10/14 (71.4%) *E. coli* isolates demonstrated ciprofloxacin resistance, and 7/14 (50%) *E. coli* isolates demonstrated resistance to TMP-SMX. Oral fosfomycin dosing regimens administered were 3 g·q 72 hours for 6 weeks in subjects 1–12 and 3 g·q 48 hours for 6 weeks in subjects 13 and 14 (Table 3). Subject 1 received a 7-day course of intravenous ertapenem prior to oral fosfomycin treatment. Clinical and microbiological treatment outcomes were documented after a median follow-up period of 20 months. Clinical cure was determined by resolution of pretreatment symptoms. Clinical failure was indicated in patients whom remained symptomatic after 2 weeks posttreatment or remained symptomatic during follow-up. Microbiological eradication was determined by a negative Meares–Stamey test or 2 negative semen cultures at 1 month and 6 months posttreatment. Microbiological eradication was observed in 8/14 (57.1%) subjects, and clinical cure was reported in 7/14 (50%) subjects (Table 3). Microbiological eradication was reported in 4/5 (80%) subjects with infection caused by MDR *E. coli*. No adverse reactions to fosfomycin therapy were reported.

Grayson et al. outlined two cases of bacterial prostatitis treated successfully with oral fosfomycin [24]. Subject 1, a 73-year-old diabetic man developed signs/symptoms including high fever, dysuria, and frequency shortly after undergoing a transrectal ultrasound- (TRUS-) guided biopsy of the prostate. The patient was diagnosed with acute prostatitis. After failing first-line antimicrobial therapy, the patient was transferred to Austin Health in Melbourne, Australia, where urine culture and prostate biopsy were performed. ESBL-producing *E. coli* was isolated in culture that demonstrated in vitro resistance to ciprofloxacin and susceptibility to meropenem, ertapenem, and fosfomycin (MIC, 1 *μ*g/mL). The biopsy showed evidence of focal acute and chronic prostatitis. Meropenem at a dose of 1 g was administered intravenously q 8 hours for 2 weeks followed by outpatient intravenous ertapenem 1 g OD for 4 weeks. Two weeks posttreatment the patient relapsed. ESBL-producing *E. coli* with the same susceptibility profile as observed previously grew in urine culture. The patient was placed back on intravenous meropenem (1 g·q 8 h) for two weeks and followed up with oral fosfomycin 3 g OD for 14 days. The fosfomycin dose was increased to 3 g BID; however, the patient displayed fecal urgency/diarrhea beginning 36 hours after the increase in dosage. The increased dose was discontinued after five days, and the patient reverted back to a 3 g OD dosage regimen. The patient completed a total course of 16 weeks oral fosfomycin. Microbiological eradication and clinical cure were documented after a 6 month follow-up period. Subject 2, an 80-year-old man was initially treated for a urinary tract infection caused by ESBL-producing *E. coli.* The susceptibility profile indicated resistance to ciprofloxacin but susceptibility to fosfomycin (MIC, 1 *μ*g/mL). The patient was treated with oral fosfomycin 3 g every 72 hours for 2 weeks. Five days posttreatment, the patient relapsed and presented with symptoms of dysuria, polyuria, and malodorous urine. A urine culture grew the same pathogen as previously identified. Computed tomography (CT) indicated an enlarged prostate. A clinical diagnosis of acute prostatitis was made, and the patient was placed back on oral fosfomycin 3 g OD for 12 weeks. Microbiological eradication and clinical cure were documented after a 6 month follow-up period.

Cunha et al. reported a single case of a 53-year-old man successfully treated for chronic prostatitis with a combination of doxycycline and oral fosfomycin [22]. The patient presented with symptoms including dysuria, frequency, and malodorous urine. Urinalysis indicated high-grade pyuria and mucous threads. Urine culture isolated ESBL-producing *E. coli* resistant to doxycycline (MIC, >16 *μ*g/mL) and fluoroquinolones (MIC, >8 *μ*g/mL) but susceptible to fosfomycin (MIC, 2 *μ*g/mL). The patient was initially treated with nitrofurantoin (100 mg·q 12 h) for one month. Nitrofurantoin treatment was unsuccessful, and the patient's symptoms persisted. Doxycycline likewise provided no improvement. The patient was then placed on oral fosfomycin 3 g·q 72 h for 1 month. Within days of initiating oral fosfomycin, the patient relapsed and symptoms returned. Urine culture grew the same pathogen as previously isolated. The patient was then treated with a high-dose course of oral fosfomycin (6 g·q 72 h for 1 month); however, the patient again relapsed. Finally, combination therapy with oral fosfomycin 3 g·q 72 h and oral doxycycline 100 mg·q 12 hours for 2 weeks resulted in sustained microbial eradication and clinical cure.

Karaiskos et al. reported on 20 patients with chronic prostatitis treated with oral fosfomycin at Hygeia General Hospital's outpatient infectious diseases clinic in Athens, Greece [25]. Of the 20 subjects (mean age of 53.6 years), *E. coli* was the causative pathogen in 13 (65%) subjects. Susceptibility data indicated that 8/13 (61.5%) *E. coli* isolates were fluoroquinolone-resistant, 7/13 (53.8%) were TMP-SMX-resistant, and 2/13 (15.4%) were ESBL producers. All isolates were susceptible to fosfomycin. Oral fosfomycin was administered at a dose of 3 g OD for one week followed by 3 g·q 48 hours for 6 weeks. The patient follow-up occurred at 3 months and 6 months posttreatment. Clinical cure was documented in 10/13 (77%) subjects. Of the three clinical treatment failures, two patients relapsed and one patient discontinued treatment due to severe diarrhea. Five of 20 subjects (25%) reported adverse effects during treatment, most commonly diarrhea.

## 7. Place in Therapy

The rapid global spread of MDR ESBL-producing *E. coli* represents a growing challenge in the treatment of acute and chronic prostatitis [3, 4]. High rates of resistance to empirical agents have consequently increased the likelihood of clinical treatment failures in cases of bacterial prostatitis, especially those caused by ESBL-producing MDR *E. coli* [7, 21]. The necessity for effective alternative oral therapies in the treatment of acute and chronic prostatitis is imperative. Fosfomycin has emerged as a potential oral therapy candidate due to superior in vitro activity (99.2% susceptibility) versus various *E. coli* resistant genotypes/phenotypes and adequate penetration into prostatic tissue (≥4 *μ*g/g in 70% of patients) [8, 9].

Current data suggests that oral fosfomycin demonstrates clinical cure rates in the range of 50–77% in patients with bacterial prostatitis caused by antimicrobial-resistant *E. coli* [7, 25]. Reported microbiological eradication rates in these same patients are >50% [7]. Effective oral fosfomycin treatment doses for chronic prostatitis have ranged from 3 g OD to 3 g·q 72 hours [7, 22, 24, 25]. Published evidence suggests that oral fosfomycin dosing frequency exceeding 3 g OD is not recommended due to the increased propensity for gastrointestinal adverse effects [10, 24]. High-dose fosfomycin (>3 g per dose) has not demonstrated improved clinical efficacy versus 3 g doses [22]. Based upon the current available evidence, an oral fosfomycin dosage of 3 g q 24 h for the first week of treatment followed by 3 g q 48 h for the remaining duration of treatment appears effective. This dosage regimen has demonstrated the highest clinical cure rates while minimizing gastrointestinal adverse effects. If gastrointestinal adverse effects occur, the fosfomycin dosage/frequency should be adjusted appropriately. Based upon the available evidence, oral fosfomycin treatment durations of 6–16 weeks appeared to be effective. We note that the majority of clinical cures have been documented after 6-7 weeks of treatment with oral fosfomycin. We are unaware of any data treating patients with bacterial prostatitis with oral fosfomycin for longer than 16 weeks. Clearly, additional clinical data are needed to determine optimal dosage/duration of oral fosfomycin treatment for acute and chronic prostatitis caused by antimicrobial-resistant *E. coli*. In addition, combination antimicrobial data with fosfomycin are required to fully assess fosfomycin's potential for treatment. In conclusion, we report that oral fosfomycin may be a reasonable treatment alternative for acute and CBP caused by antimicrobial-resistant *E. coli.* In addition, oral fosfomycin may also be appropriate for use when first-line treatments fail or cannot be used due to adverse effects.

## Figures and Tables

**Table 1:** In vitro activities of orally prescribed antimicrobial agents against urine isolates of *E. coli* collected by 15 clinical laboratories across Canada from 2007 to 2015^a^.

*E. coli* phenotype^b^ (*n*)	Antimicrobial agent	CLSI MIC interpretation^f^		
		% *S*	% *I*	% *R*
All *E. coli* (1,207)	Fosfomycin	99.2	0.7	0.1
	AMC^c^	87.1	9.6	3.3
	Ciprofloxacin	81.1	0	18.9
	TMP-SMX^d^	75	—	25
TMP-SMX-resistant (302)	Fosfomycin	99.7	1.3	1
	AMC	74.8	22.2	3
	Ciprofloxacin	57.6	0	42.4
	TMP-SMX	0	0	100
Ciprofloxacin-resistant (228)	Fosfomycin	96.1	0.4	3.5
	AMC	71.5	22.8	5.7
	Ciprofloxacin	0	100	100
	TMP-SMX	43.9	—	56.1
ESBL-positive (61)	Fosfomycin	95.1	4.9	0
	AMC	63.9	32.8	3.3
	Ciprofloxacin	16.4	0	83.6
	TMP-SMX	34.4	—	65.6
AMC-resistant (40)	Fosfomycin	100	0	0
	AMC	0	0	100
	Ciprofloxacin	67.5	0	32.5
	TMP-SMX	77.5	—	22.5
Resistant to TMP-SMX and CIP^e^ (128)	Fosfomycin	97.7	0.7	1.6
	AMC	66.4	28.9	4.7
	Ciprofloxacin	0	0	100
	TMP-SMX	0	0	100
Resistant to CIP and AMC (13)	Fosfomycin	100	0	0
	AMC	0	0	100
	Ciprofloxacin	0	0	100
	TMP-SMX	53.8	—	46.2
Resistant to TMP-SMX and AMC (9)	Fosfomycin	100	0	0
	AMC	0	0	100
	Ciprofloxacin	33.3	0	66.7
	TMP-SMX	0	0	100
Multidrug-resistant (12)	Fosfomycin	100	0	0
	AMC	16.7	33.3	50
	Ciprofloxacin	0	0	100
	TMP-SMX	0	0	100

^a^Data adapted from reference [4]; ^b^ESBL, extended-spectrum *β*-lactamase; multidrug-resistant was defined as isolates resistant to ≥3 agents from different antimicrobial classes (amoxicillin-clavulanate, ciprofloxacin, nitrofurantoin, and TMP-SMX); ^c^AMC, amoxicillin-clavulanate; ^d^TMP-SMX, trimethoprim-sulfamethoxazole; ^e^CIP, ciprofloxacin; ^f^based on CLSI fosfomycin MIC breakpoints for *E. coli*: susceptible, ≤64 *μ*g/mL; intermediate, 128 *μ*g/mL; and resistant, ≥256 *μ*g/mL (CLSI, M100-S26, 2016).

**Table 2:** Fosfomycin concentrations in plasma, urine, and prostate following a single 3 g oral dose^a^.

Parameter	Mean (±SD)	Median	Range
Plasma			
Time after dose (min)	565 (149)	578.5	385–995
Concentration (*μ*g/mL)	11.42 (7.60)	10.84	2.29–40.38
Urine			
Time after dose (min)	581 (150)	593	398–1020
Concentration (*μ*g/mL)	570.57 (418.40)	434.86	47.99–1522.05
Prostate transition zone			
Time after dose (min)	598 (152)	598	420–1025
Concentration (*μ*g/g)	8.30 (6.63)	5.35	0.56–26.05
Prostate peripheral zone			
Time after dose (min)	608 (155)	598	420–1067
Concentration (*μ*g/g)	4.42 (4.10)	2.97	0.17–18.06
Prostate mean			
Time after dose (min)	602.87 (153.36)	598	420–1046
Concentration (*μ*g/g)	6.50 (4.93)	4.67	0.67–22.06
Prostate/plasma ratio	0.67 (0.57)	0.50	0.07–2.92

^a^Data adapted from reference [9]; SD, standard deviation.

**Table 3:** Fosfomycin (FOS) treatment for acute and chronic bacterial prostatitis caused by antimicrobial-resistant *E. coli*.

Reference	Subject number	Type of bacterial prostatitis^a^	Age	Susceptibility		FOS MIC	MDR^d^ mechanism	FT^ e^ dosage regimen	FT treatment duration	Microbiological eradication	Clinical cure
				CIP^b^	TMP-SMX^c^						
[7]	1	C	80	R^g^	R	—	AmpC	3 g·q 72 h	6 weeks	Yes	Yes
	2	C	52	R	R	—	ESBL^i^	3 g·q 72 h	6 weeks	Yes	Yes
	3	C	54	R	S	—	ESBL	3 g·q 72 h	6 weeks	No	No
	4	C	54	R	R	—	—	3 g·q 72 h	6 weeks	No	No
	5	C	31	R	S	—	—	3 g·q 72 h	6 weeks	No	No
	6	C	29	S^h^	S	—	ESBL	3 g·q 72 h	6 weeks	Yes	Yes
	7	C	57	R	R	—	—	3 g·q 72 h	6 weeks	Yes	Yes
	8	C	22	R	R	—	—	3 g·q 72 h	6 weeks	Yes	Yes
	9	C	44	S	R	—	—	3 g·q 72 h	6 weeks	No	No
	10	C	59	R	S	—	ESBL	3 g·q 72 h	6 weeks	Yes	Yes
	11	C	49	R	R	—	—	3 g·q 72 h	6 weeks	Yes	No
	12	C	70	R	S	—	—	3 g·q 72 h	6 weeks	No	No
	13	C	65	S	S	—	—	3 g·q 48 h	6 weeks	No	No
	14	C	54	S	S	—	—	3 g·q 48 h	6 weeks	Yes	Yes
[24]	1	A/C	73	R	—	1 *μ*g/mL	ESBL	3 g OD/BID	16 weeks	Yes	Yes
	2	A	80	R	—	1 *μ*g/mL	ESBL	3 g OD	12 weeks	Yes	Yes
[22]	1	C	53	R	—	2 *μ*g/mL	ESBL	3 g·q 72 h^∗^	2 weeks	Yes	Yes
[25]	13 total	C	53.6^f^	8/13 R	7/13 R	13/13 S	2/13 ESBL	3 g OD q 48 h	7 weeks	—	∼77%

^a^A, acute prostatitis; C, chronic prostatitis; U, unspecified; ^b^CIP, ciprofloxacin; ^c^TMP-SMX, trimethoprim-sulfamethoxazole; ^d^MDR, multidrug-resistant; ^e^FT, fosfomycin tromethamine; ^f^mean age (years); ^g^R, resistant; ^h^S, susceptible; ^i^ESBL, extended-spectrum *β*-lactamase; ^j^MBL, metallo-*β*-lactamase; ^∗^combination therapy of oral FT (3 g q 72 h) and oral doxycycline (100 mg q 12 h).
